# Exploring the Potential Impact of Nanoparticles on Fetal Development: An Updated Review

**DOI:** 10.3390/medicina62081599

**Published:** 2026-08-20

**Authors:** Romualdo Sciorio, Federica Cariati, Othman F. Abdelzaher, Mohammed Adel, Gyongyver Teglas, Carlo Alviggi, Steven Fleming

**Affiliations:** 1 Vitrolife-Group, 421 32 Västra Frölunda, Sweden; 2 Department of Public Health, School of Medicine, University of Naples Federico II, 80131 Naples, Italy; 3 Zoology Department, Faculty of Science, Al-Azhar University, Cairo 11884, Egypt; 4 Department of Reproductive Medicine and Gynecology Endocrinology, Lucerne University Cantonal Hospital, 6004 Lucerne, Switzerland; 5 Discipline of Anatomy & Histology, School of Medical Sciences, University of Sydney, Sydney, NSW 2006, Australia

**Keywords:** nanoparticles, embryonic development, placental transfer, pregnancy outcomes, oxidative stress, fetotoxicity

## Abstract

Nanomaterials are increasingly used in manufacturing, medicine, consumer products, and environmental technologies due to their unique physicochemical properties. Although these materials offer substantial technological and societal benefits, their widespread use has raised concerns about potential health risks. Of particular importance is exposure during pregnancy, as certain nanoparticles can cross the placental barrier and reach the developing embryo. Fetal tissues are highly sensitive to environmental insults, so maternal exposure to nanoparticles may disrupt normal development and increase the risk of abnormal pregnancy outcomes. This review examines the current understanding of nanoparticle-induced developmental toxicity, with a focus on the vulnerability of the maternal–fetal unit. We discuss the structure and function of the placental barrier and the mechanisms that enable nanoparticle transfer from mother to fetus. Particular attention is given to how nanoparticle characteristics, including size, shape, composition, and surface chemistry, influence biodistribution, placental transport, tissue accumulation, and toxicity. We summarize the major molecular and cellular mechanisms implicated in fetotoxicity, highlighting oxidative stress, apoptosis, autophagy, and DNA damage as recurring pathways identified across experimental studies. These interconnected processes contribute to placental dysfunction, impaired fetal growth, developmental abnormalities, and adverse pregnancy outcomes. We also compare findings across different classes of nanoparticles, including metal, metal oxide, carbon-based, and polymeric nanomaterials, identifying both shared toxicological mechanisms and material-specific effects. Evidence from animal models demonstrates that susceptibility varies according to nanoparticle properties, exposure conditions, and species, underscoring the complexity of nanoparticle–biological interactions and the limitations of extrapolating experimental findings directly to humans. Overall, the available evidence indicates that nanoparticle exposure during pregnancy represents a potential risk to fetal health, although important knowledge gaps remain regarding human exposure and long-term developmental outcomes. A better understanding of the mechanisms underlying nanoparticle-induced fetotoxicity is essential for improving human health risk assessment, refining experimental models, informing regulatory policies, and supporting the safe-by-design development of nanomaterials. Such knowledge will help ensure the responsible application of nanotechnology while minimizing potential risks during pregnancy. Finally, this review is distinguished by its integrated analysis of how the chemical characteristics of nanoparticles govern placental transfer and the mechanistic pathways of fetotoxicity across multiple nanomaterial classes, providing a unified framework that connects material properties with their potential for abnormal fetal development and adverse pregnancy outcomes.

## 1. Introduction

**Figure 1:**
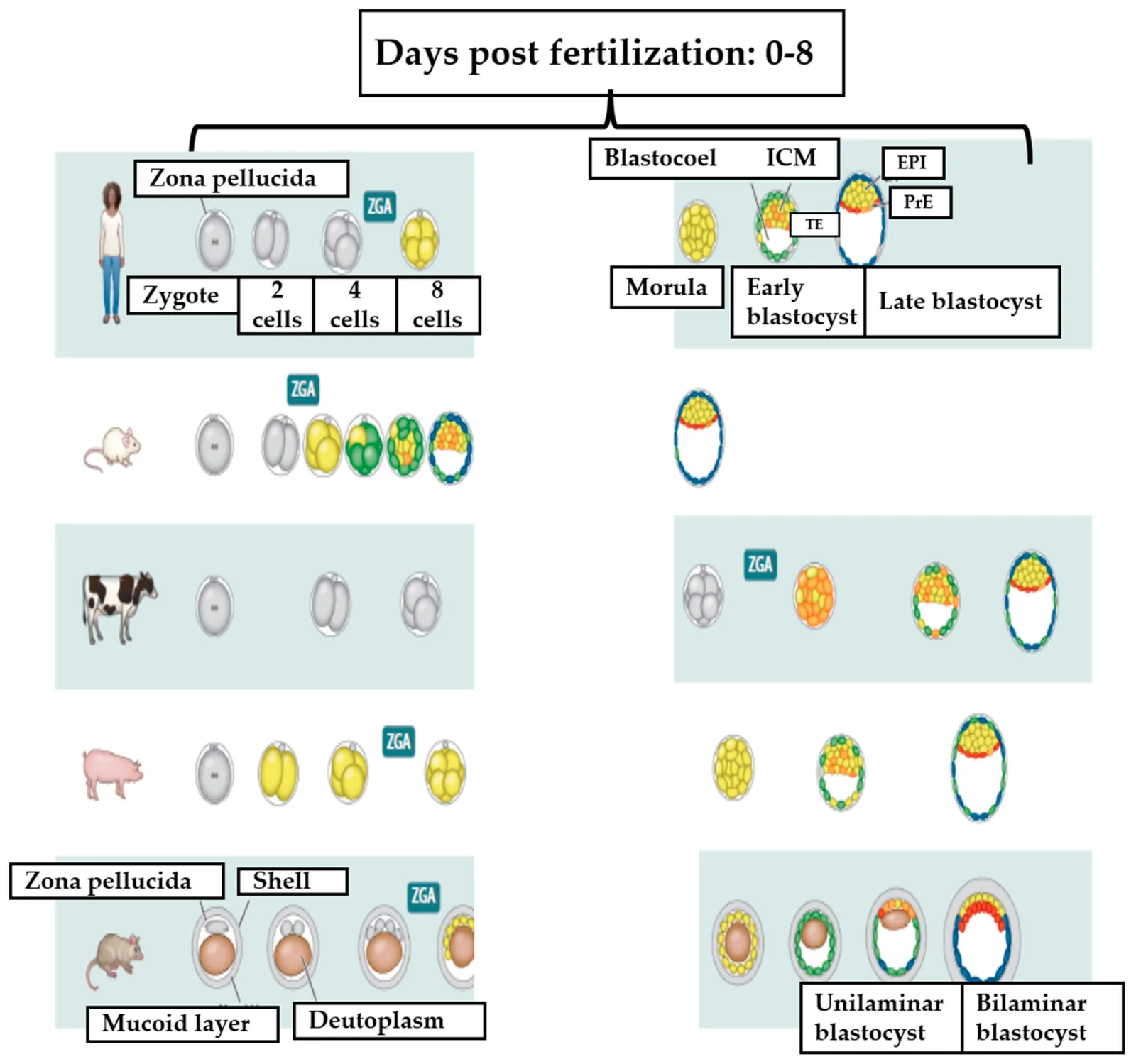
Comparison of preimplantation development from fertilization to blastocyst formation in human, mouse, cow, pig, and opossum embryos. Embryos are aligned along a temporal axis beginning with the zygote and progressing through the cleavage stages, morula, and blastocyst, highlighting the timing of cell fate specification events [13]. EPI: epiblast; ICM: inner cell mass; PrE: primitive endoderm; TE: trophectoderm; ZGA: zygotic genome activation.

Nanoparticles (NPs) are ultrafine materials with dimensions ranging from 1 to 100 nm [1]. At this scale, matter exhibits unique optical, electrical, and physicochemical properties that differ markedly from those of bulk materials. These characteristics have facilitated their widespread application in medicine, industry, and environmental technologies, including their use in imaging and drug delivery systems that allow real-time monitoring of cellular uptake and biological functions [1,2]. Despite these advantages, increasing evidence indicates that NPs may pose risks to both human and environmental health. The magnitude of these risks depends on individual susceptibility as well as intrinsic characteristics of NPs, including size, shape, chemical composition, surface charge, and surface coatings [2]. A major concern associated with exposure to NPs is their ability to cross biological barriers that typically protect sensitive tissues. Studies have demonstrated that certain NPs can traverse the blood–brain, blood–testis, and blood–placental barriers, leading to their accumulation in vulnerable organs [3]. These properties raise concerns regarding reproductive health. The female reproductive system, especially during pregnancy, is highly sensitive to xenobiotics due to dynamic hormonal regulation and extensive tissue remodelling [4]. Nanoparticles may enter the body through inhalation, ingestion, or dermal absorption and subsequently reach the systemic circulation, enabling their distribution to reproductive organs and the developing fetus. Exposure to NPs during critical windows of prenatal or gestational development has been associated with developmental toxicity and an increased risk of congenital abnormalities [5]. Because reproductive tissues undergo continuous physiological and structural changes, even minor disruptions can interfere with normal embryonic and fetal development [6]. Environmental pollutants, including engineered nanomaterials, have been shown to adversely affect maternal health and fetal outcomes, prompting the use of diverse experimental models to investigate reproductive and developmental toxicity [7]. During pregnancy, NPs may cross the placental barrier and reach fetal organs, where immature detoxification and defence mechanisms render the developing embryo particularly vulnerable to toxic insults. Consequently, reproductive toxicity has emerged as a critical component of NP risk assessment [8]. Recent reviews emphasize that reproductive and developmental toxicity remain insufficiently characterized aspects of nanomaterial safety, highlighting the need for integrated experimental, epidemiological, and translational approaches to better assess fetal risk during pregnancy [6,7,8]. Notably, exposure during gametogenesis—even at the single-gamete level—may have lasting consequences for fertility and offspring health [9]. Nanoparticles may exert toxic effects on embryos either directly, by inducing malformations, growth retardation, or embryonic lethality, or indirectly, through maternal toxicity, inflammation, or oxidative stress [10]. Experimental studies have shown that NPs can cross the blood–testis and blood–follicle barriers and accumulate in gonadal tissues, including ovarian cells, potentially impairing reproductive function [11]. Moreover, gold (Au), silver (Ag), and silica NPs have been demonstrated to cross the placental barrier in pregnant mice following intravenous or oral exposure, underscoring the relevance of these findings for human health [12]. Early embryonic development begins with fertilization and the formation of a diploid zygote, followed by rapid cleavage divisions that generate blastomeres without an increase in total embryonic volume (Figure 1). These stages culminate in compaction, morula formation, and subsequent cavitation to form the blastocyst [13]. Unlike previous reviews that focus primarily on placental transfer or postnatal toxicity, the present work integrates placental biology, molecular mechanisms, and organ-specific developmental outcomes to provide a comprehensive framework for understanding NP-induced fetotoxicity.

## 2. Placental Barrier Structure and Mechanisms of Nanoparticle Transfer

**Figure 2:**
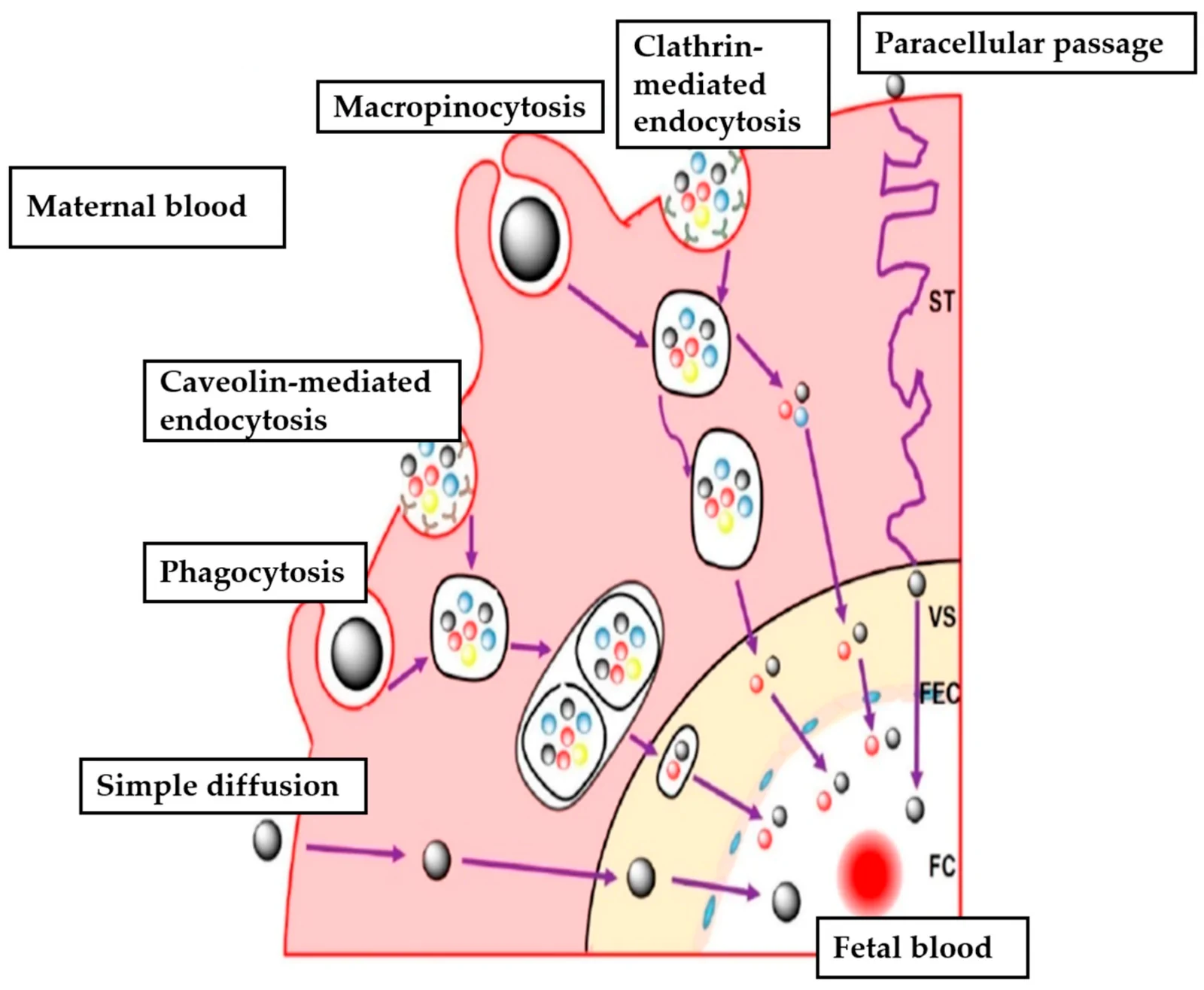
Schematic representation of the potential fetotoxic effects of various nanoparticles (NPs). The physicochemical properties of NPs, such as size, shape, composition, and surface chemistry, play a crucial role in determining their fetotoxic potential following maternal exposure during pregnancy. ST: Syncytiotrophoblast; VS: Villous stroma; FEC: Fetal endothelial cells; FC: Fetal capillary.

The placenta constitutes the physiological interface between mother and fetus, regulating the exchange of nutrients, gases, and waste products while also acting as a selective barrier to xenobiotics, including NPs [14]. Structurally, the human placenta is a hemochorial organ composed of chorionic villi containing fetal capillaries enclosed by a continuous syncytiotrophoblast layer, with an underlying cytotrophoblast layer that becomes discontinuous as gestation progresses [15]. These trophoblast layers form the principal interface for maternal–fetal exchange and regulate the transport of nutrients, gases, xenobiotics, and NPs. In contrast, rodent placentae are also hemochorial but exhibit a hemotrichorial organization characterized by three trophoblast layers separating maternal and fetal blood. This additional trophoblast complexity, together with species-specific differences in placental architecture, transporter expression, vascular organization, and gestational development, influences barrier permeability and the transplacental transfer of xenobiotics [16]. Consequently, although rodent models are invaluable for investigating mechanisms of developmental toxicity, differences in placental structure and function should be carefully considered when extrapolating NP biodistribution and fetal exposure data to humans. Placental transfer is primarily regulated by trophoblast and fetal endothelial cells, whose tight junctions and selective transport systems restrict paracellular diffusion while controlling the passage of macromolecules and NPs [17]. As a result, NPs predominantly cross the placental barrier via intracellular pathways, including passive diffusion, active transport, and endocytosis [18]. Experimental studies demonstrate that the translocation of NPs is strongly dependent on particle size, composition, and surface properties. For example, quantum dots and silica or titanium dioxide (TiO_2_) NPs below ~150 nm can accumulate in placental and fetal tissues, whereas larger particles are largely excluded [19]. Endocytosis mechanisms, including clathrin- and caveolin-mediated pathways (Figure 2), have been implicated in the maternal–fetal transfer of several nanomaterials, such as Au and zirconia NPs [20]. Very small NPs may also exploit transtrophoblastic channels, while paracellular diffusion is considered unlikely for particles larger than 10 nm [20,21]. Importantly, NP transfer is not consistent but varies across gestation, as placental thickness, vascularization, and transport capacity change during pregnancy [21]. Recent advances using human-relevant placental models, including ex vivo placental perfusion systems and placenta-on-a-chip platforms, have provided more physiologically accurate insights into NP transfer kinetics and maternal–fetal barrier function, improving translational relevance compared with traditional animal models [19,20,21]. Nevertheless, although rodents remain indispensable experimental models for developmental nanotoxicology, important structural and functional differences between rodent and human placentae should be considered when extrapolating experimental findings to human pregnancy. These interspecies differences may influence NP transport, placental accumulation, and fetal exposure, thereby affecting the interpretation and clinical translation of toxicological data.

## 3. Oxidative Stress, Inflammation, and Genotoxic Effects

**Figure 3:**
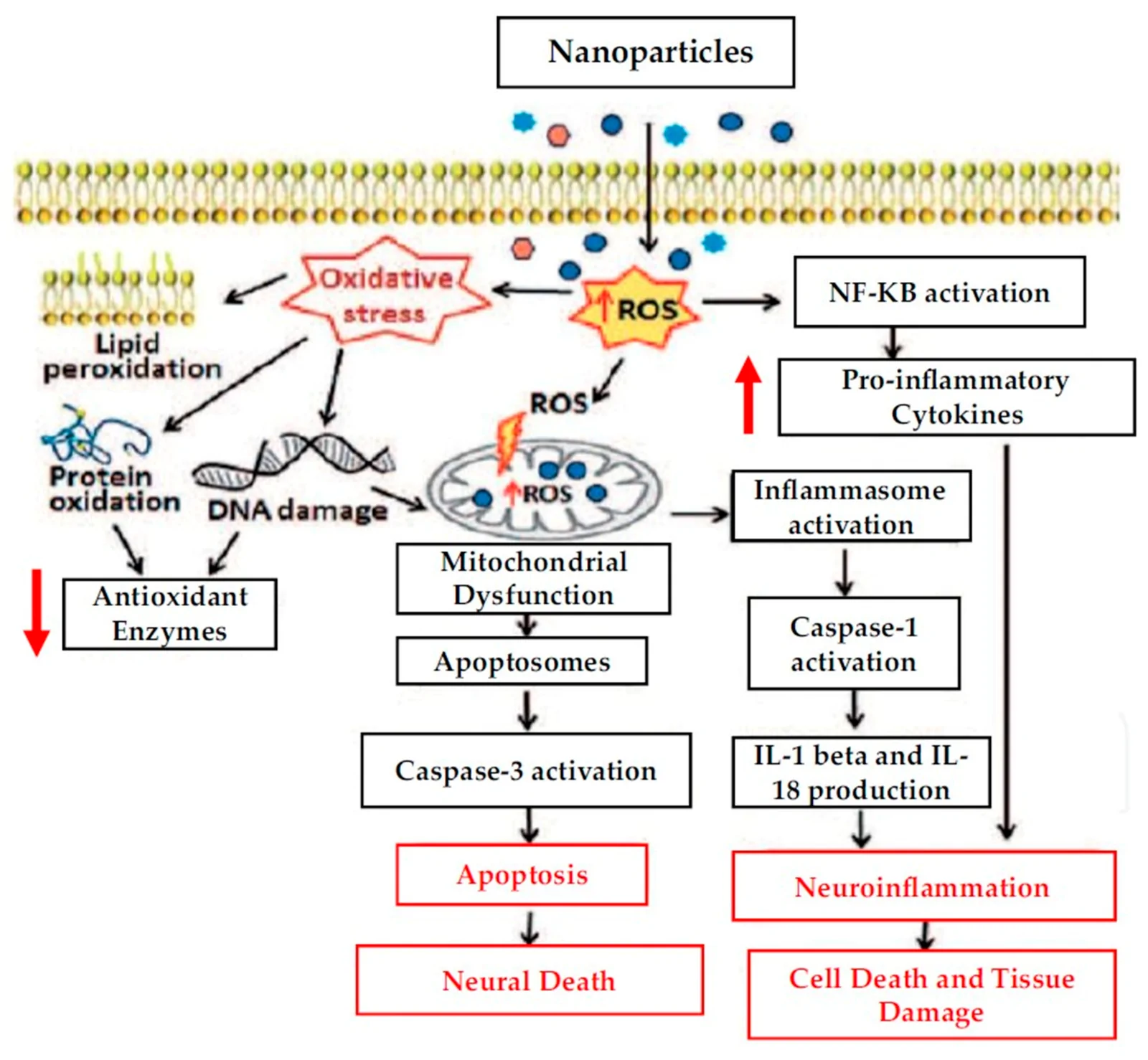
Mechanisms of nanoparticle (NP)-induced neurotoxicity. NPs increase reactive oxygen species (ROS) production, causing oxidative damage to lipids, proteins, and mitochondrial and nuclear DNA. ROS impair antioxidant enzymes and promote mitochondrial dysfunction, leading to intrinsic apoptotic cell death in neurons. Mitochondrial damage also activates inflammasomes, stimulating caspase-1-dependent release of IL-1β and IL-18.

Oxidative stress and inflammation are widely recognized as central mechanisms underlying NP-induced developmental and fetotoxicity [10]. Prenatal exposure to carbon black and TiO_2_ NPs has been shown to induce reactive oxygen species (ROS) generation, lipid and DNA oxidation (Figure 3), and astrocyte activation in the brains of offspring [22]. Inhaled NPs can also elicit maternal pulmonary inflammation, indirectly impairing fetal development, as demonstrated following TiO_2_ NP exposure during mid-gestation (gestational day 8–18) [12]. Once internalized by placental cells, excessive ROS production promotes placental inflammation and dysfunction, contributing to pregnancy complications. Increased oxidative stress has been observed in malformed fetuses and placentae following single-wall carbon nanotube exposure [23], while ROS-mediated downregulation of vascular endothelial growth factor has been linked to placental vascular impairment, miscarriage, and fetal growth restriction [24]. Recent mechanistic studies further confirm that oxidative stress acts as a central driver of NP-induced developmental toxicity through redox-sensitive signalling pathways and inflammatory cascades [22]. In parallel, NPs can directly induce genotoxic effects, including DNA strand breaks, mutations, deletions, and oxidative DNA adduct formation during pregnancy [25]. Maternal inhalation of carbon black NPs resulted in DNA strand breaks in offspring tissues [26], while TiO_2_ NP exposure altered retinoic acid pathway gene expression in a sex-specific manner. Size-dependent clastogenic and epigenetic alterations have been reported following gold (Au) NP exposure during organogenesis, and diesel exhaust particles induced DNA deletions in developing tissues [27]. Notably, cobalt–chromium NPs exacerbated hippocampal DNA damage through autophagy dysregulation and IL-6-mediated inflammatory signalling [28]. Emerging evidence further suggests that prenatal exposure to NPs may lead to persistent epigenetic modifications, raising concerns regarding long-term and potentially transgenerational effects on reproductive and developmental health [19,20,21].

## 4. Apoptosis and Autophagy

**Figure 4:**
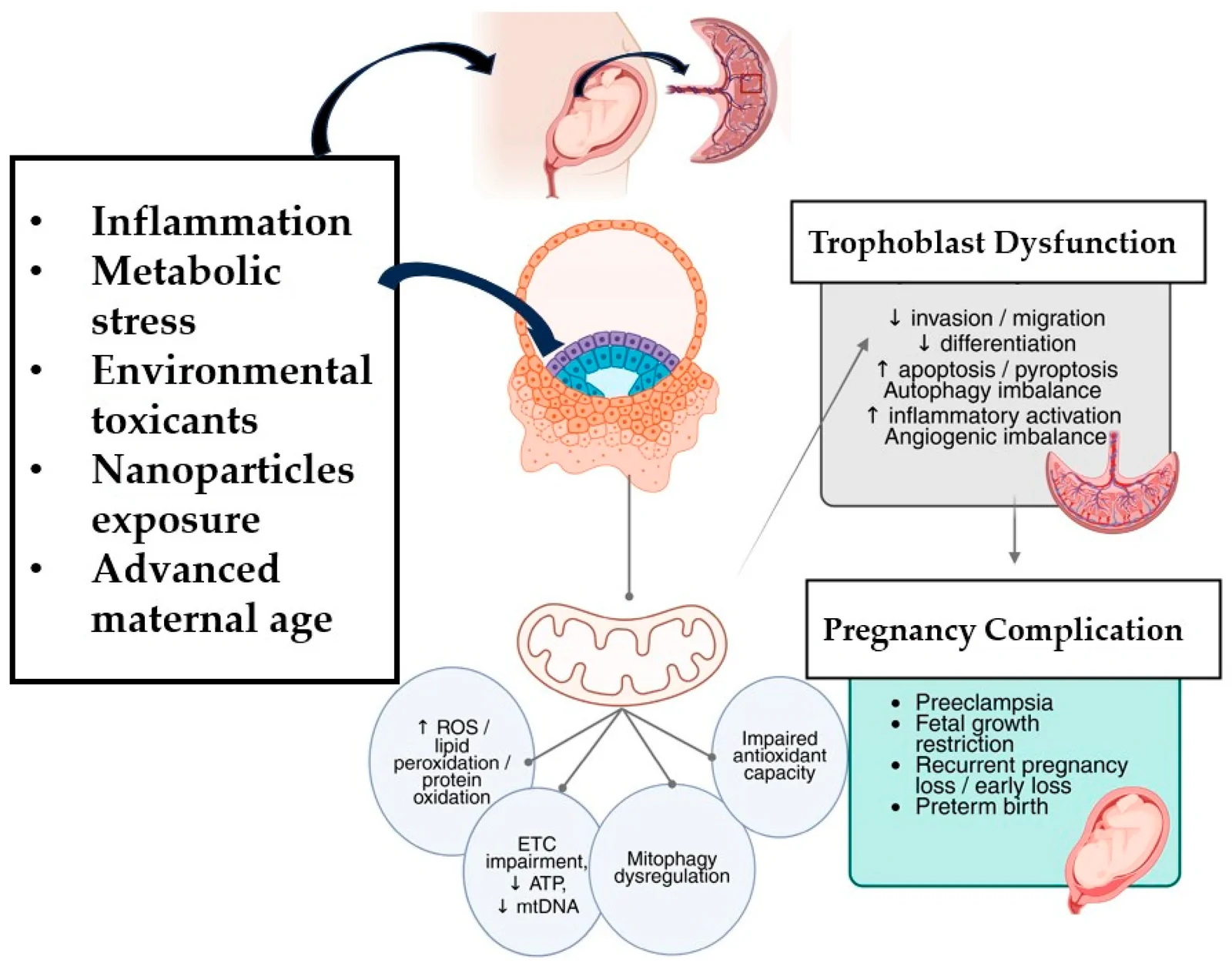
Trophoblast mitochondrial dysfunction, driven by metabolic stress, exposure to nanoparticles, environmental toxicants, and advanced maternal age, impairs ATP production, damages mtDNA, and increases ROS. Resulting oxidative stress and inflammation disrupt trophoblast function, reducing placental development and increasing risks of miscarriage, fetal malformations, and pregnancy complications. Abbreviations: ROS, reactive oxygen species; ETC, electron transport chain; mtDNA, mitochondrial DNA; ATP, adenosine triphosphate; ↑, increased; ↓, decreased.

Gestational exposure to various nanomaterials, including silica dioxide (SiO_2_), TiO_2_, and Au NPs, quantum dots, carbon nanotubes, , and polystyrene beads, has been shown to induce apoptosis in placental spongiotrophoblasts, highlighting direct placental toxicity [29,30]. Apoptotic responses have also been documented in the offspring brain following maternal exposure to ultrafine NPs, suggesting transplacental or indirect neurotoxic effects [31]. Mechanistically, functionalized multi-walled carbon nanotubes (MWCNTs) trigger p53-dependent apoptosis and cell cycle arrest through DNA damage pathways [25] while carboxylate-modified polystyrene beads, as well as SiO_2_, zirconium dioxide (ZrO_2_), and Au NPs, have been shown to cross the placental barrier and disrupt placental apoptotic signalling [29,32]. In parallel, increasing evidence indicates that NPs can modulate or disrupt autophagy, a process essential for oocytogenesis, implantation, placentation, and embryogenesis [33,34]. NP-induced autophagic alterations have been reported for MWCNTs and molybdenum disulfide (MoS_2_) nanosheets in a manner dependent on particle size, surface chemistry, and structural properties [35]. Experimental studies have demonstrated that cobalt–chromium (CoCr) NPs impair autophagic flux in a BeWo model (BeWo is a human placental cell line widely used as an in vitro model of the placental barrier) leading to altered neural progenitor differentiation and DNA damage in derived neurons and astrocytes [28]. It was originally derived from a human placental choriocarcinoma (a cancer of trophoblast cells), but despite its origin, it has become one of the standard laboratory models for studying placental transport and function. Excessive autophagy triggered by TiO_2_ NPs has also been associated with disrupted dendritic development in the hippocampus of exposed offspring [36]. Collectively, these findings indicate that dysregulated autophagy and apoptosis act in concert as critical mechanisms linking NP exposure to impaired placental development and adverse neurodevelopmental outcomes. Essentially, NPs can have both direct and indirect impacts upon normal placental and foetal physiological function and development, ultimately leading to increased risk of miscarriage and foetal malformation (Figure 4). Direct effects of NPs include disruption of autophagy and vascular impairment of the placenta, chromosomal mutations, deletions and rearrangements, as well as epigenetic alterations to the maternal and embryonic genome. Indirect effects of NPs are driven primarily by ROS, with NP-induced ROS causing oxidative stress which can result in lipid peroxidation and mitochondrial dysfunction. Lipid peroxidation can result in DNA oxidation, oxidative DNA adduct formation, autophagy, apoptosis, ferroptosis, necroptosis and pyroptosis. Mitochondrial dysfunction can result in production of mitochondrial ROS via oxidative phosphorylation and may lead to inflammatory responses mediated by inflammasomes, Caspase I, IL-1β, IL-6 and IL-18. Redox-sensitive signalling pathways can also mediate p53-dependent apoptosis and cell cycle arrest. Hence, NPs can be embryotoxic and may disrupt the normal physiological apoptotic signalling and inflammatory responses within the placenta, leading to complications of pregnancy.

## 5. Ferroptosis

Oxidative stress, induced by increased levels of ROS, can activate various pathways of programmed or regulated cell death including apoptosis, necroptosis and ferroptosis. Essentially, ferroptosis is a non-apoptotic, intracellular iron-catalysed, widespread and lethal level of lipid peroxidation that occurs due to the aggregation of ROS and iron ions [37]. Ferroptosis may also be induced by an intracellular iron overload via a non-canonical pathway [38]. The balance between iron accumulation-induced ROS and the antioxidant system within a cell determines whether ferroptosis occurs. Reduction of lipid peroxides by scavengers such as Coenzyme Q10, which is abundant within the cell membrane, can offer some protection against ferroptosis [39]. Ferroptosis may also be prevented by the glutathione-dependent lipid repair hydroperoxidase, glutathione peroxidase 4 (GPX4), which reduces lipid hydroperoxides into non-toxic lipid alcohols and is the major mechanism by which cell membranes protect themselves against peroxidative damage [40]. Conversely, ferroptosis may be induced either by inactivation of GPX4 or by increasing the labile iron pool. Inactivation of GPX4 may be achieved by inhibition of the cystine/glutamate antiporter system, xc^−117^, which deprives GPX4 of its cofactor, glutathione. The xc^−^ system is composed of a regulatory subunit solute carrier family 3 member 2 (SLC3A2) and a catalytic subunit solute carrier family 7 member 11 (SLC7A11). Indeed, deliberate activation of ferroptosis within ferroptosis-sensitive cells using anti-tumour nanodrug delivery systems has been implemented clinically to intentionally treat various forms of cancer [41]. These nanodrugs act by increasing intracellular iron ion content and suppressing GPX4 to elevate intracellular ROS and accumulate lipid peroxides [42]. Magnetic iron oxide (Fe_3_O_4_) particles may be used for such a purpose, though they are typically used as contrast agents during magnetic resonance imaging. For example, an external magnetic field has been used in conjunction with magnetic Fe_3_O_4_ nanotubes loaded with the small molecule ferroptosis inducer, RSL3, which directly inhibits GPX4, to actively target liver tumour cells without any cytotoxic effect upon normal hepatic cells [43]. In addition to ferroptosis, magnetic Fe_3_O_4_ particles can generate ROS, leading to oxidative stress, inflammation, genotoxicity and apoptosis, and their targeting efficiency in vivo is often low [44]. Intravenous injection of ferroptosis-inducing magnetic Fe_3_O_4_ NPs is commonly used in biomedical applications and, therefore, their biodistribution, biotransformation, bioactivity and clearance from the body should be considered, particularly during pregnancy. In this respect, ferroptosis has been reported within umbilical endothelial cells following exposure to iron-bearing NPs.

## 6. Effects of Nanoparticles upon Embryogenesis in Mammalian Models

Embryogenesis is characterized by rapid and tightly regulated organ formation, rendering the developing embryo particularly vulnerable during implantation, placentation, and organogenesis [45]. Rodent models, particularly mice and rats, are extensively used to investigate the embryotoxic and teratogenic effects of environmental contaminants because of their well-characterized development, short gestation, and physiological similarities to humans [46]. Increasing evidence indicates that engineered NPs can cross the placental barrier and accumulate in both embryonic and extra-embryonic tissues, raising concerns about their potential to disrupt normal fetal development [19]. Although the extent of NP transplacental transfer differs among mammalian species owing to variations in placental architecture, transporter expression, and gestational physiology, studies consistently demonstrate that NPs can reach the developing embryo. Furthermore, while placental maturation may reduce translocation efficiency during late gestation by strengthening barrier function, fetal exposure still occurs and has been associated with developmental abnormalities, growth restriction, oxidative stress, and altered organogenesis in experimental animal models [47,48,49]. Experimental evidence shows that SiO_2_ and TiO_2_ NPs can penetrate embryonic tissues and exert teratogenic effects [14]. Quantum dots have been reported to induce embryonic necrosis and yolk sac and tail malformations, effects not solely attributable to released cadmium ions (Cd^2+^), suggesting nanoscale-specific mechanisms of toxicity [50]. Moreover, maternal exposure to TiO_2_ NPs impaired offspring neurobehavior and reproductive capacity, with gene expression analyses revealing alterations in inflammatory and immune pathways, supporting indirect placental- or maternal-mediated mechanisms even in the absence of detectable accumulation of NPs within the fetus [51].

### 6.1. Developmental Toxicity of Engineered Nanoparticles

Engineered NPs differ markedly in physicochemical properties, exposure routes, and biological behaviour, resulting in heterogeneous patterns of developmental toxicity. Nevertheless, accumulating evidence from in vitro and in vivo models indicates that multiple classes of NPs can interfere with embryonic development, placental function, and postnatal organ maturation through shared mechanisms, including oxidative stress, inflammation, apoptosis, and barrier disruption.

### 6.2. Silver Nanoparticles

Silver NPs are widely used for their antimicrobial properties [52,53]. In vitro, Ag NPs induce apoptosis in both trophectoderm (TE) and the inner cell mass (ICM) of mouse blastocysts, significantly inhibiting proliferation [54]. In vivo, blastocyst transfer after Ag NP exposure revealed impaired development [54]. Intravenous Ag NP exposure during late pregnancy altered vascular contractility in a size-dependent manner [55], while nasal inhalation increased fetal resorptions [56]. Repeated injections of 10 nm Ag NPs led to maternal accumulation in the liver, spleen, and visceral yolk sac, with limited fetal accumulation but with potential impacts on embryonic growth [57]. Ag NPs impaired offspring spatial learning and memory [58] and maternal exposure caused hippocampal structural abnormalities in offspring [59]. Ag NPs were also found in offspring tissues, suggesting placental or lactational transfer [60].

### 6.3. Titanium Dioxide Nanoparticles

**Figure 5:**
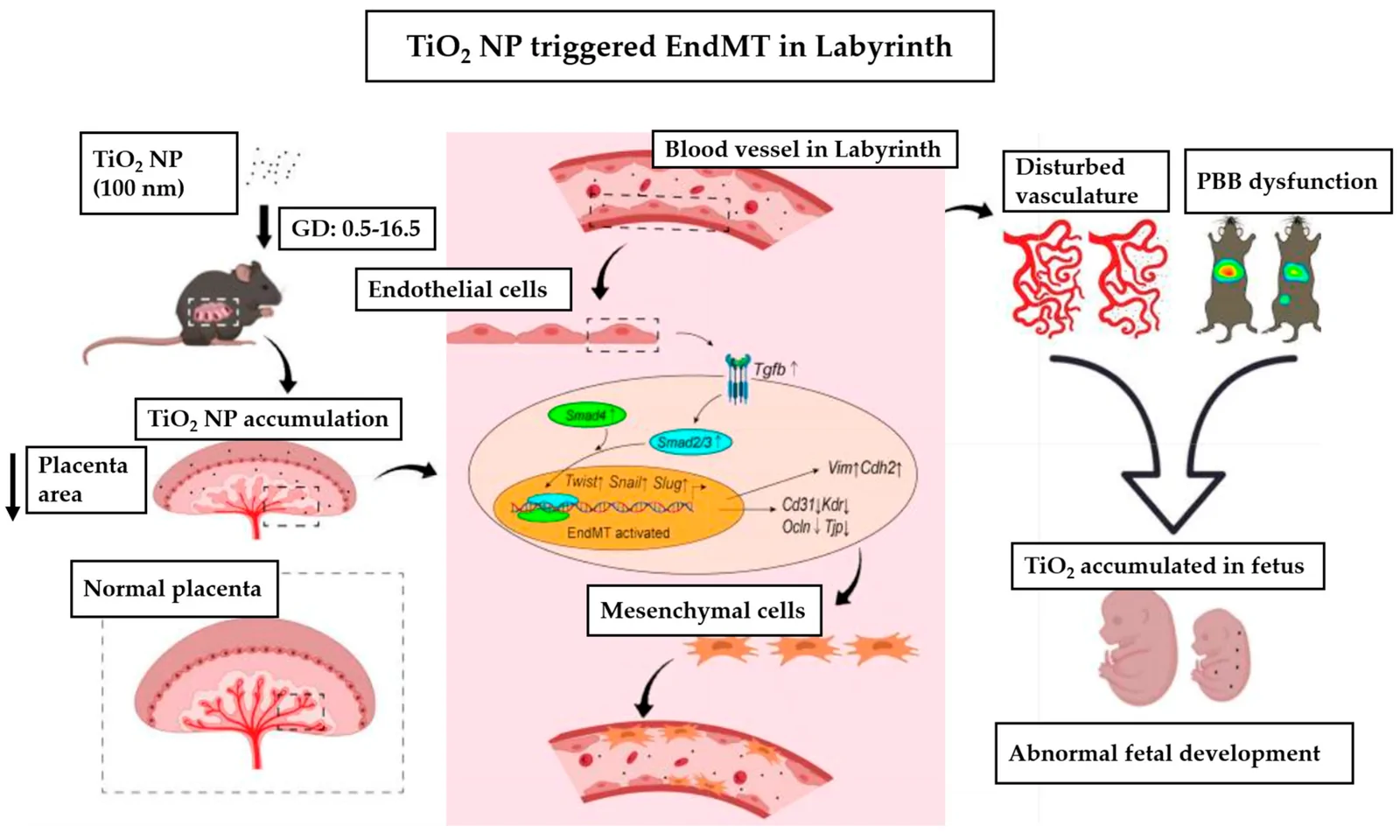
Mechanisms underlying titanium dioxide (TiO_2_) nanoparticle (NP)-induced embryonic and fetal developmental toxicity. Maternal exposure to 100 nm TiO_2_ NPs caused placental accumulation and fetal transfer, inducing endothelial-to-mesenchymal transition (EndMT) in the placental labyrinth, impairing endothelial function, disrupting the placental barrier, and resulting in fetal growth restriction. GD: Gestational Day; PBB: Placental blood barrier.

TiO_2_ NPs are widely applied in consumer products [61] and have well-documented reproductive and developmental toxicity. Chronic intragastric exposure reduced offspring number in a dose-dependent manner. TiO_2_ NPs induced morphological, skeletal, and organ (brain, liver, kidney) abnormalities in exposed litters [62] as well as pregnancy complications linked to structural and functional abnormalities within the placenta [14]. TiO_2_ NPs can cross both the blood–placental and blood–brain barriers, accumulating in fetal brains and impairing dendritic and axonal development [36]. They also dysregulate genes involved in development of the central nervous system (CNS) [63]. Toxicity depends on NP size, crystalline form, and illumination; the mineral form of TiO_2_, anatase, is more reactive than rutile [64]. Exposure to TiO_2_ NPs induced sperm defects, cranial nerve abnormalities, memory impairment, reduced hippocampal proliferation, and behavioural deficits [65,66,67]. Inhalation caused persistent maternal lung inflammation and neurobehavioral changes in offspring [51]. Prenatal exposure altered brain regions including olfactory bulb, cerebral cortex, and dopaminergic circuits [68]. TiO_2_ exposure also affected retinoic acid pathway gene expression in the liver of offspring [26], that may trigger endothelial-to-mesenchymal transition (EndMT), impairing placental vascularization [69,70,71,72]. Developmental toxicity includes neurotoxicity, immunotoxicity, reproductive and respiratory defects (Figure 5).

### 6.4. Zinc Oxide Nanoparticles

Zinc oxide (ZnO) NPs are widely used in consumer products [73]. They can cross the placental barrier and accumulate in the fetal liver [74]. High doses increased fetal resorption, reduced the number of live pups, and lowered fetal weights [75]. ZnO NPs induced teratogenicity, such as yolk sac abnormalities and impaired morphology [76], together with oxidative-stress-related molecular changes (upregulation of caspase-3; downregulation of cytoplasmic superoxide dismutase and phospholipid hydroperoxide glutathione peroxidase). Surface charge influenced toxicity: positively charged particles caused fetal abnormalities, while negatively charged ones did not [77].

### 6.5. Cadmium-Based Nanoparticles

Cadmium (Cd) NPs are widely used in the production of galvanic cells, ceramics, pigments for paints and plastics, and in electro-galvanization; cadmium–nickel alloys are essential for accumulators and batteries, fertilizers, cigarettes, X-ray screens and kinescopes [78]. Cd NPs also arise as by-products during the refining of copper, lead and zinc, contributing to long-standing environmental contamination through wastewater, fertilizers and atmospheric emissions, through which they enter the food chain and accumulate in humans [79]. In women, Cd exposure may cause hormonal dysregulation, impaired ovulation and fertilization, spontaneous abortion, gestational toxicosis and congenital abnormalities [80]. Cd NPs are embryotoxic, genotoxic and teratogenic in several species; maternal exposure disrupts placental function and nutrient transport through ROS and reactive nitrogen species generation , inducing oxidative stress and macromolecular damage [81]. During pregnancy, both inhalational and oral Cd absorption increase due to physiological adaptations such as elevated respiratory rate, reduced gastrointestinal motility and delayed gastric emptying [82]. Cd NPs accumulate in lung or gut depending on exposure route and are subsequently distributed to liver, kidneys, placenta, mammary glands, uterus and fetus; they can also be excreted into milk [83]. Table 1 and Table 2 summarize the key structural and functional differences among human, mouse, and rat placentae and their implications for translating NP toxicity studies to human pregnancy, alongside published studies investigating the effects of NPs on embryogenesis.

## 7. Fetotoxicity of Nanoparticles: Mechanisms and Organ-Specific Effects

### 7.1. Neurotoxicity

Nanoparticles are capable of crossing the blood–brain barrier (BBB), a highly selective interface that normally restricts the entry of xenobiotics into the CNS [26,51,103,104]. During pregnancy, maternal exposure to engineered or environmental NPs is of particular concern because these particles may also cross the placental barrier and the immature fetal BBB, increasing the susceptibility of the developing brain to toxic insults. Experimental studies have demonstrated that prenatal exposure to ultrafine particles and TiO_2_ NPs induces structural and functional alterations in the fetal brain, including neuronal necrosis, dysregulated gene expression, and persistent behavioral abnormalities in offspring [105,106,107,108,109]. Proposed mechanisms include oxidative stress, neuroinflammation, impaired autophagy, lysosomal dysfunction, and mitochondrial damage. In addition, prenatal TiO_2_ NP exposure has been associated with altered dopamine metabolism in the prefrontal cortex and striatum, suggesting long-lasting effects on neurotransmitter homeostasis and neurodevelopment [104,105,107,108,109,110,111,112,113].

### 7.2. Reproductive Toxicity

Maternal NP exposure reduces testis and epididymis weight in offspring and causes structural seminiferous tubule damage and reduced daily sperm production [111,112,113]. Diesel exhaust particles disrupt testicular development, decrease accessory gland weights and increase sperm DNA fragmentation [113,114] . Oxidative stress is considered one of the principal mechanisms underlying NP-induced male reproductive toxicity and has been implicated in approximately 30–80% of male infertility cases. Excessive production of ROS disrupts the balance between oxidants and antioxidants, resulting in lipid peroxidation, DNA damage, mitochondrial dysfunction, apoptosis, and impaired spermatogenesis. Experimental studies have shown that gestational exposure to carbon nanoparticles induces persistent alterations in the seminiferous epithelium that extend into adulthood. Similarly, exposure to ZnO, TiO_2_, Fe_2_O_3_, and SiO_2_ NPs adversely affects testicular structure and function, leading to reduced sperm quality and infertility [114,115,116,117]. TiO_2_ NPs preferentially accumulate in Sertoli cells, Leydig cells, and developing germ cells, causing seminiferous tubule degeneration, endocrine dysfunction, and impaired sperm production, thereby highlighting the potential long-term reproductive risks associated with NP exposure [115,116,117,118].

## 8. Recent Advances in Nanotoxicology

Recent advances in experimental models are providing more physiologically relevant tools to investigate NP-induced developmental toxicity and overcome many of the limitations associated with the application of in vitro systems and animal models. Compared with traditional static in vitro culture, these models more accurately reproduce placental architecture, nutrient exchange, barrier function, and the mechanical forces experienced during in vivo processes. They enable quantitative assessment of NP translocation, cellular uptake, and toxic responses under conditions that more closely mimic human physiology [119,120,121]. Placenta-on-chip platforms have emerged as a promising feature for investigating nanomaterial toxicity by recreating the dynamic maternal–fetal interface through the integration of trophoblast and endothelial cell layers under controlled microfluidic flow. One such organ chip microdevice comprises a collagen IV-coated cell culture insert seeded with TE-derived human trophoblast stem cells and co-cultured above human umbilical vein endothelial cells, creating a placental bilayer separated by the culture insert porous membrane [120]. This microdevice was placed on a culture plate rocker to simulate continuous fluid flow. Once the trophoblast stem cells had differentiated into cytotrophoblast and syncytiotrophoblast, thereby capitulating the trophoblastic bilayered epithelium of placental villi, NPs were added from above to simulate maternal blood exposure. Following 48 h exposure to 20 μg.mL^−1^ of copper oxide (CuO) NPs (40 nm), further differentiation of the trophoblast cells was inhibited, as was their secretion of human chorionic gonadotrophin. Furthermore, the CuO NPs disrupted autophagic flux within the trophoblast and induced apoptosis, with evidence of an inflammatory response including macrophage activation, inflammatory cytokine secretion, and endothelial barrier disruption. Interestingly, TiO_2_ NPs (20 μg.mL^−1^; 25 nm) demonstrated no detrimental impact in this microdevice [120]. However, in another placenta-on-chip model, higher concentrations (50 μg.mL^−1^ and 200 μg.mL^−1^) of TiO_2_ NPs were found to generate ROS, cause trophoblast cell death, and impair placental barrier function, which was associated with attraction of maternal macrophages by the trophoblast cells [122]. Like placenta-on-chip models, multicellular engineered living systems such as microphysiological systems and organoids have been developed to investigate reproductive biology and toxicology [123,124,125]. These models more faithfully recapitulate trophoblast differentiation, endocrine activity, and cell–cell interactions than conventional two-dimensional cultures, providing valuable platforms for investigating the mechanisms underlying NP-induced placental toxicity. Given the vast array of NPs that have been approved for clinical usage and considering the diversity of cell types within the body, it is hardly surprising that conventional methods for assessing nanotoxicology within tissues often prove to have limited precision and accuracy. To complicate matters further, NPs may have complex interactions with both intracellular and extracellular compartments within various tissues. Fortunately, the concept of functional fingerprinting addresses this issue since integrated biological responses to NPs may be revealed via interdisciplinary transcriptomic, proteomic, metabolomic, lipidomic, multi-omic, and emerging single-cell or spatial approaches [126,127,128,129,130,131,132,133]. In parallel, single-cell RNA sequencing (scRNA-seq), spatial transcriptomics, and integrated multi-omics approaches are transforming mechanistic toxicology by enabling the identification of cell type-specific molecular responses, intercellular communication networks, and coordinated changes in gene expression, protein abundance, metabolism, and epigenetic regulation following NP exposure. Together, these technologies provide unprecedented spatial and molecular resolution for elucidating how distinct placental cell populations contribute to impaired placental function, abnormal fetal development, and adverse pregnancy outcomes, while allowing us to better understand the cellular and subcellular compartments involved, and the varied and complex mechanism of action of NPs upon cell viability [126,127,128,129,130]. Furthermore, functional fingerprinting enables correlations to be made between a cell’s genotype and phenotype. Most importantly, the fingerprint imparted by a NP helps predict its therapeutic efficacy relative to its potential toxicity within a given biological and environmental context, even at low exposure levels. Higher-resolution single-cell transcriptomics enable differentiation between heterogenous cell responses to NPs whereas spatial transcriptomics preserves tissue architecture, as well as biological and environmental context. The application of functional fingerprinting to the impact of NPs during conception and pregnancy should prove invaluable. However, the big data generated by functional fingerprinting requires complicated statistical analysis using bioinformatics tools, and this is where artificial intelligence (AI)-assisted nanotoxicology may prove beneficial. In this respect, deep learning can assist in predicting interactions between NPs and biological systems, whereas machine learning predicts the physicochemical properties and biological interactions of NPs via analysis of their structural features [134]. The intersection between functional fingerprinting and AI-assisted nanotoxicology has led to the concepts of safe-by-design and sustainable-by-design smart nanomaterials [133]. These computational approaches facilitate the development of robust quantitative structure–activity relationship (nano-QSAR) models capable of identifying toxicity patterns, prioritizing safer nanomaterials for experimental testing, and reducing reliance on animal studies. Combined with physiologically based pharmacokinetic modeling and high-content omics datasets, AI-driven platforms can improve the prediction of maternal and fetal exposure while supporting mechanistic interpretation of toxicological responses [129,130,131,132,133,134].

## 9. Conclusions and Future Perspectives

Although the toxicity of nanomaterials during pregnancy has been widely investigated, more systematic and comprehensive studies are still needed. Significant knowledge gaps remain that must be addressed to improve risk assessment and ensure their safe use. To date, most studies on NP-induced developmental toxicity have focused on the nervous, reproductive, respiratory, and immune systems. However, future research should expand to investigate other vulnerable targets, including cardiac development, fetal metabolism, and endocrine function, which may also be susceptible to maternal NP exposure. Equally important is a deeper understanding of placental biology, particularly the mechanisms governing early placental development and the timing and pathways through which NPs cross the maternal–fetal interface. Further studies are also needed to systematically elucidate how the physicochemical properties of NPs, including size, shape, surface charge, coating, composition, solubility, and aggregation state, influence their biodistribution, placental transfer, and toxicity. Establishing clear structure–activity relationships will be essential for predicting biological behavior and supporting the rational design of safer nanomaterials. In addition, important uncertainties remain regarding both the short- and long-term consequences of prenatal NP exposure. Well-designed experimental and epidemiological studies are therefore required to evaluate fetal development, postnatal health, and the potential for transgenerational effects. To conclude, nanomaterials are now widely incorporated into cosmetics, medical devices, pharmaceuticals, textiles, construction materials, and numerous consumer products, increasing the likelihood of human exposure. Consequently, assessing their ability to cross placental barriers and induce developmental toxicity has become a public health priority. Current evidence indicates that maternal NP exposure can adversely affect offspring by inducing neurotoxicity, reproductive toxicity, immunotoxicity, and respiratory toxicity, with these outcomes being strongly influenced by the physicochemical characteristics of the NPs as well as the nature, dose, and timing of maternal exposure. Therefore, standardized strategies for environmental and occupational exposure assessment are urgently needed to establish scientifically grounded safety thresholds. Collectively, the available evidence supports a shift from descriptive toxicological studies toward mechanism-based, placenta-centered safety assessment frameworks that integrate placental function with developmental endpoints. Such approaches, combined with safe-by-design principles and harmonized regulatory strategies, are essential for developing nanomaterials that are both innovative and safe for maternal and fetal health. This review advances this objective by integrating NP physicochemical properties, biodistribution, placental transport, and the molecular mechanisms of developmental toxicity across diverse nanomaterial classes. By identifying both shared and material-specific pathways of fetotoxicity, it provides a comprehensive framework to improve risk assessment, refine experimental models, and support the rational design of safer nanomaterials.

## Figures and Tables

**Table 1:** Comparison of key structural and functional differences among human, mouse, and rat placentae and their implications for translating nanoparticle toxicity studies to human pregnancy.

Characteristic	Human	Mouse	Rat	Implications for Nanoparticle Studies	References
**Placental architecture**	Villous hemochorial	Labyrinthine hemochorial	Labyrinthine hemochorial	The maternal–fetal exchange region is organized differently among species; this should be considered when comparing placental nanoparticle distribution and transplacental transfer.	[3,15,84,85,86]
**Maternal-fetal barrier**	Hemomonochorial	Hemotrichorial	Hemotrichorial	Differences in trophoblast barrier organization may contribute to model-dependent nanoparticle uptake and transplacental passage.	[3,84,85,86]
**Trophoblast differentiation**	Villous cytotrophoblasts differentiate into syncytiotrophoblast and extravillous trophoblast	Differentiation into trophoblast giant cells, spongiotrophoblast, labyrinth trophoblast, and syncytiotrophoblast	Differentiation into trophoblast giant cells, spongiotrophoblast, glycogen cells, invasive trophoblast, and syncytial trophoblast	Species-specific trophoblast differentiation generates distinct placental cell populations that may interact differently with nanoparticles and affect their movement across the placental barrier.	[87,88,89]
**Invasive trophoblast**	Extensive extravillous trophoblast invasion	Limited trophoblast invasion	Endovascular and interstitial trophoblast invasion	Species differences in trophoblast invasion should be considered when interpreting placental findings from rodent nanoparticle studies.	[86,89]
**Spiral artery remodeling**	Extensive trophoblast- and maternal leukocyte-associated remodeling	Predominantly uterine natural killer cell-dependent	Sequential uterine natural killer cell- and invasive trophoblast-directed remodeling	The cellular mechanisms of uteroplacental vascular remodeling differ among species and should be considered when interpreting vascular or perfusion-related placental findings after nanoparticle exposure.	[85,86,87,88,89,90]
**Endocrine function**	Placental production of human chorionic gonadotropin, progesterone, estrogens, corticotropin-releasing hormone, placental lactogen, and placental growth hormone	Placental lactogens; continued ovarian dependence for progesterone and estrogen production; no placental corticotropin-releasing hormone or aromatase	Placental endocrine activity mainly in trophoblast giant cells and the junctional zone; continued ovarian contribution to steroidogenesis; no placental corticotropin-releasing hormone or aromatase	The location and regulation of hormone production differ substantially among species; endocrine or developmental effects observed after nanoparticle exposure in rodents may therefore not reproduce human placental endocrine responses.	[86,88,90]
**Maternal-fetal immune interface**	Coordinated interactions among trophoblasts, decidual leukocytes, and stromal cells support maternal tolerance and host defense	Conserved trophoblast–immune cell interactions with species-specific immune composition and regulation	Uterine natural killer cells interact with invasive trophoblasts and participate in uterine vascular remodeling	Species-specific immune regulation may modify placental inflammatory and vascular responses to nanoparticle exposure.	[87,88,89,90,91]
**Maternal-fetal nanoparticle transfer**	Demonstrated in human in vitro and ex vivo models; transfer varies with particle characteristics and experimental model	Demonstrated in vivo; transfer varies with particle properties and experimental conditions	Demonstrated in vivo and ex vivo; transfer varies with particle properties and experimental conditions	Nanoparticle transfer is particle- and model-dependent. Evidence obtained in rodents should therefore be interpreted together with human-relevant placental models rather than used as a direct quantitative estimate of human fetal exposure.	[3,86,89,90,91,92]

**Table 2:** Summary of published studies investigating the effects of nanoparticles on embryogenesis.

Nanoparticle	Particle Size	Experimental Model	Dose/Concentration	Exposure Route	Exposure Window	Main Reported Effect	Reference
Ag NPs	~13 nm	Mouse blastocysts	25 and 50 µM	In vitro culture exposure	Blastocyst stage	Induced apoptosis in both the inner cell mass (ICM) and trophectoderm (TE), reduced blastocyst cell number, impaired implantation, increased post-implantation embryonic resorption, and decreased foetal and placental weight.	[54]
Ag NPs	20 nm and 110 nm	Pregnant rats	700 μg/kg	Intravenous, tail vein	Gestational Days (GD): 17–19; assessed 24 h post-exposure	Acute intravenous Ag NP exposure induced particle size- and vehicle-dependent alterations in uterine vascular contractility. Citrate-stabilized Ag NPs increased uterine artery contractility, suggesting potential impairment of uteroplacental blood flow and foetal growth.	[55]
Ag NPs	18–20 nm	Pregnant mice	640 µg/m^3^; 3.80 × 10^7^ particles/cm^3^	Nasal inhalation	GD 0.5–14.5	Ag NPs crossed the placental barrier, accumulated in placenta and foetal tissues, increased foetal resorptions and placental inflammation following maternal inhalation exposure.	[56]
Ag NPs	10 nm	Pregnant mice	66 μg Ag/mouse (2.2 mg Ag/kg b.w.)	Intravenous, tail vein	GD 7, GD 8 and GD 9; assessed at GD 10 and GD 16	Maternal and yolk-sac accumulation; potential impairment of embryo growth	[57]
Ag NPs	32 ± 6.6 nm (SEM; size distribution 5–70 nm)	Prenatally exposed mouse offspring	0, 0.2 and 2 mg/kg	Subcutaneous injection	Every 3 days from GD 3 until delivery	Prenatal Ag NP exposure impaired spatial learning and memory and induced neurobehavioural abnormalities in adult offspring, with more pronounced effects at the higher dose.	[58]
Ag NPs	7.9 ± 0.95 nm	Pregnant rats	250 mg/kg	Oral administration	14 days before mating, during mating, throughout gestation, and until 4 days postpartum	Ag NPs crossed the placental barrier and accumulated in the liver, kidney, lung and brain of offspring.	[60]
nSP70 (silica NPs) and TiO_2_ NPs	nSP70: 70 nm; TiO_2_: 35 nm	Pregnant mice	0.8 mg/mouse	Intravenous injection, tail vein	GD 16-GD 17; evaluated on GD 18	Both nSP70 and TiO_2_ NPs crossed the placenta and accumulated in the placenta, fetal liver and fetal brain, causing fetal resorption, fetal growth restriction and placental dysfunction.	[14]
CuO, TiO_2_ and ZnO NPs	CuO: 34.4 nm; TiO_2_: 47.0 nm; ZnO: 66.9 nm	*Xenopus laevis* embryos	10, 100, 500 mg/L	Aqueous exposure: FETAX (Frog Embryo Teratogenesis Assay–Xenopus)	Mid-blastula stage (Stage 8) to 96 h post-fertilization (hpf)	CuO NPs showed the highest teratogenic potential with severe malformations and growth retardation; ZnO NPs caused marked intestinal lesions and growth retardation; TiO_2_ NPs induced mild embryotoxicity with significantly increased malformations but little effect on mortality or growth.	[93]
Surface-coated TiO_2_ NPs (UV-Titan L181)	20.6 nm	Pregnant mice	42 mg/m^3^, 1 h/day	Inhalation	GD 8–18	Prenatal inhalation induced persistent maternal lung inflammation and moderate neurobehavioural alterations in adult offspring, without major effects on gestational or litter parameters.	[94]
TiO_2_ NPs (Aeroxide P25)	21 nm	Pregnant rats	11.7 ± 1.0 mg/m^3^, 6 h/day	Inhalation	GD 11–16	Maternal inhalation exposure increased placental vascular resistance, impaired endothelium-dependent placental and umbilical artery vasodilation, increased angiotensin II sensitivity, and compromised placental hemodynamics.	[94]
TiO_2_ NPs	<25 nm	Pregnant rats	0.5 mg/kg/day	Intraperitoneal injection	From GD5 through end of lactation	Reduced fetal weight and placental weight, increased fetal resorption, growth retardation, morphological and skeletal malformations, histopathological changes in the fetal brain, liver and kidney, and increased oxidative stress.	[62,95,96]
TiO_2_ NPs	~21 nm	Pregnant rats	20 mg/mL suspension, 0.1 mL/10 g body weight/day	Oral gavage	GD 6–12	TiO_2_ NPs were detected in maternal and neonatal lungs, inducing pulmonary inflammatory lesions and delayed saccular development in neonatal offspring.	[95]
Surface-coated TiO_2_NPs (UV-Titan L181)	17 nm	Pregnant mice	42 mg/m^3^, 1 h/day	Inhalation	GD 8–18	Sex-specific alterations in offspring hepatic gene expression (retinoic acid signalling), without increased DNA strand breaks.	[94]
TiO_2_ NPs	100 nm	Pregnant mice	10, 50 and 250 mg/kg/day	Oral gavage	GD 0.5–16.5	TiO_2_ NPs crossed the placental barrier, impaired placental vascular development through endothelial-to-mesenchymal transition (EndMT), disrupted placental barrier function, and caused dose-dependent fetal growth restriction	[96]
TiO_2_ NPs	12–14, 80–100 and 150–200 nm	Zebrafish embryos	20 ppt	Aqueous exposure	2.5–72 hpf	Size-dependent developmental toxicity: incomplete notochord formation, heart defects (heart muscle atrophy, pericardial edema and blood pooling), epidermal inflammation and altered expression of immune response, Tumor Necrosis (TNF) and endocytosis-related genes, with the strongest effects observed for 12–14 nm TiO_2_ NPs.	[97]
TiO_2_ NPs	6.5 nm	Pregnant mice	1, 2 and 3 mg/kg BW/day	Oral gavage	GD 7–21	Maternal TiO_2_ NP exposure inhibited hippocampal dendritic outgrowth and induced oxidative stress, mitochondrial dysfunction, apoptosis and excessive autophagy in offspring neurons.	[98]
Mesoporous silica and ZnO NPs	*NR*	Pregnant mice	ZnO NPs: 50–300 mg/kg BW; bulk ZnO: 100 mg/kg BW; silica nanoparticles (SN): 50–250 mg/kg BW	Oral gavage	GD15–GD19 (alternate days)	High-dose ZnO NPs induced miscarriage and fetal loss. Prenatal ZnO NP and silica nanoparticles exposure altered steroidogenic gene expression and caused testicular histopathological changes in male offspring, whereas SNs showed lower reproductive toxicity than ZnO NPs.	[99]
ZnO NPs	30 nm	Zebrafish embryos	1, 5, 10, 25, 50, 100 mg/L	Aqueous exposure	3–96 hpf	Dose-dependent developmental toxicity, including delayed hatching, reduced larval body length, tail malformations, and embryonic lethality at 50–100 mg/L	[100]
ZnO NPs	30 ± 10 nm	Pregnant mice	20, 60, 180 and 540 mg/kg/day	Oral gavage	GD 10.5–17.5	Dose-dependent maternal and fetal toxicity, including fetal growth restriction, reduced fetal number, placental structural damage, and placental oxidative stress, endoplasmic reticulum stress, apoptosis and functional impairment.	[101]
ZnO NPs + CdCl_2_	14.2 ± 2.7 nm	Pregnant mice	ZnO NPs: 133.3, 200, 300 mg/kg BW/day; CdCl_2_: 8 mg/kg BW/day	Oral gavage	GD 1–10 or GD 7–16	Co-exposure increased maternal–fetal transfer, disrupted placental tight junctions, aggravated embryotoxicity, and increased fetal growth impairment, bone deformities and fetal loss, particularly after organogenesis exposure.	[102]

## Data Availability

No new data were created or analyzed in this study. Data sharing is not applicable to this article.
